# Effects of Contrast Agent and Outer Volume Saturation Bands on Water Suppression and Shimming of Hepatic Single-Volume Proton MR Spectroscopy at 3.0T

**DOI:** 10.1100/2012/804698

**Published:** 2012-11-20

**Authors:** Li Xu, Yan Huang, Xian Liu, Bo Liu

**Affiliations:** ^1^Department of Radiology, Guangdong Provincial Traditional Chinese Medicine Hospital and Postdoctoral Mobile Research Station of Guangzhou University of Traditional Chinese Medicine, 111 Da De Lu, Guangdong, Guangzhou 510120, China; ^2^Department of Neurology, Guangdong Provincial Traditional Chinese Medicine Hospital and Postdoctoral Mobile Research Station of Guangzhou University of Traditional Chinese Medicine, 111 Da De Lu, Guangdong, Guangzhou 510120, China

## Abstract

*Purpose*. To determine whether administration of gadolinium diethylenetriamine pentaacetic acid (Gd-DTPA) and whether placement of the outer volume saturation bands significantly affect shimming and water suppression on hepatic MR spectroscopic prescanning. *Method*. Region of interest (ROI) of 2 cm × 2 cm × 2 cm was carefully positioned in the region of the middle portion of the right hepatic lobe. 32 patients were examined before and after administration of Gd-DTPA with and without outer-volume saturation bands. Linewidths (Full-Width Half-Maximum (FWHM)) and water suppression were obtained. A paired *t*-test for comparison of means was used. *Results*. (1) The group with the outer volume saturation bands demonstrated slightly better water suppression effect than the group without outer volume saturation bands before administration. (2) The group with the outer volume saturation bands demonstrated better water suppression effect than the group without outer volume saturation bands after administration. (3) Both shimming and water suppression effectswere decreased on enhanced MR spectroscopic prescanning (all *P* < 0.05). *Conclusions*. Placement of the outer volume saturation bands is helpful to improve water suppression both before and after contrast agent administration. Gd-DTPA exerts a slightly adverse effect (a statistically significant but clinically unimportant) on magnetic resonance spectroscopic prescanning at 3T.

## 1. Introduction

Magnetic resonance spectroscopy (MRS) is a noninvasive technique which is being increasingly applied to delineate biochemical changes of the liver. *In vivo *proton MRS has been applied to many areas of clinical liver research, including investigations of cirrhosis, hepatitis, and diagnosis of malignancies and treatment monitoring which are still in early stages of development [1–5]. The recent installation of higher field strength (3T) clinical magnets with multicoil arrays for the body offer new opportunities for performing body MR spectroscopy. The improved SNR can reduce acquisition times and the higher field strength also provides better separation of resonances [6, 7].

The diagnostic value of abdominal MRS relies on adequate technical factors such as the prescan adjustments of shimming and effective water suppression [8]. Linewidth is usually defined as the full-width at half-maximum peak height (FWHM) in frequency domain. It determines the capability of MRS to discern spectral features. As shimming improves the field homogeneity, linewidths become smaller and the spectroscopy resolution is enhanced. Strong resonance signals in prescans from the hydrogens in water molecules may interfere the signals from the lower concentration compounds of interest. The water signal may be suppressed to better discern the resonance signals of compounds of interest [7, 9, 10].

In hepatic magnetic resonance imaging, MRS may be added to existing protocols and is usually acquired before administration of intravenous contrast agent. However, under certain circumstances, MRS after administration of contrast agent may be desirable if it can achieve equivalent results. For example, the use of ^1^H-MRS after gadolinium-enhanced MRI is a reasonable approach with practical advantages of better localization of the ROI to the enhancing area [11, 12].

In the study of the nervous system, some authors view these effects of Gd-DTPA as clinically unimportant. However, according to the data from our previous study of kidney, after administration of Gd-DTPA, statistically significant decrease in water suppression and shimming effects were noted and both effects were constantly stabilized with time extension. This phenomenon limits the diagnostic use of kidney MRS examinations performed immediately after contrast-enhanced MRI. It seems that “organ-difference” existed [7]. 

The aim of this study was to assess whether administration of gadolinium diethylenetriamine pentaacetic acid (Gd-DTPA) and whether placement of the outer volume saturation bands significantly affects shimming and water suppression on hepatic MRS prescan adjustments on a 3.0T system.

## 2. Materials and Methods

### 2.1. Subjects

 The study was approved by our institutional review board, and written informed consent was obtained from all patients. 32 patients (16 men, 16 women; range, 21–74; median age, 47 years) with no history of liver disease and with normal liver function test results to evaluate nonhepatic disease or fatty liver were included in this study.

### 2.2. Magnetic Resonance Spectroscopy Protocol (Figure 1)

The examinations were performed on a GE Signa 3.0T whole-body system (GE Signa Excite HD; GE Medical Systems, Milwaukee, Wis) with the standard proton MRS acquisition software provided by the manufacturer. The body coil was used as the transmitter, and a torso phased array coil (eight coils, four anterior and four posterior coils, Waukesha, Wis) was used as the receiver. Single-volume spin-echo point-resolved spectroscopy (PRESS) was used with parameters of 1500/30/64 (TR/TE/excitations) in all patients. The patients entered the magnet in a supine position with their feet first. Anatomical imaging was carried out at the end expiration or during trigger mode. The localizer image for the MRS Voxel was selected from the anatomical images. 

**Figure 1:**
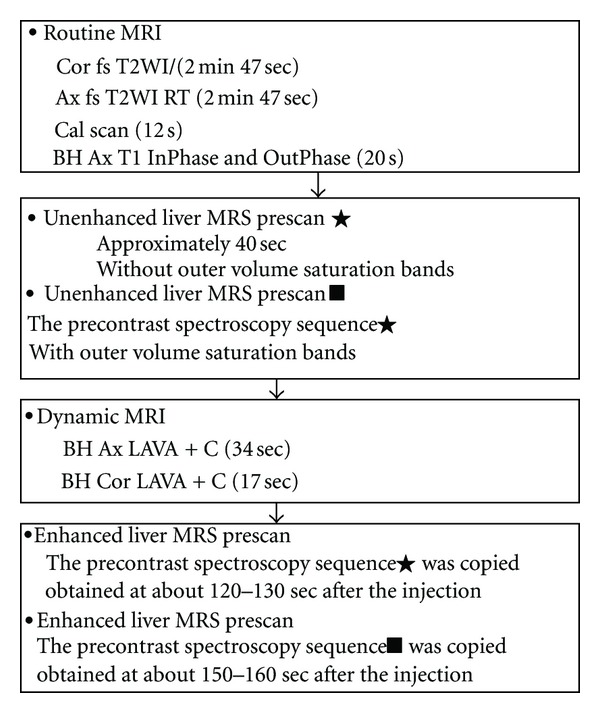
Detailed scanning protocols of routine MRI, unenhanced and enhanced MRS Prescan of shimming and water suppression. BH: breath hold; RT: respiratory triggering; LAVA: liver acquisition with volume acceleration.

A voxel of 20 × 20 × 20 mm was positioned in the right hepatic lobe, avoiding inclusion of the diaphragm and edges of the liver, but also vascular and biliary structures. The precontrast spectroscopy sequence was “copied” for the postcontrast acquisition to produce identical voxel positioning (with and without the outer volume saturation bands) between pre- and postcontrast measurements provided the patient did not move. Before administration of Gd-DTPA, the shimming and water suppression were performed. Then, Gd-DTPA (Magnevist, Bayer Schering; 0.1 mmol/kg) was injected as a rapid bolus and immediately followed by a 30 mL saline flush through a power injector at a rate of 2 mL/s. We looked for signs of motion artifact on the imaging acquisitions and performed image subtractions of the last imaging sequence at the end of the study from the initial imaging sequence (obtained just before spectroscopic measurements) to see if the patient may have shifted in position during the examination. Patients who exhibited motion were excluded in this study. One patient was excluded for analysis because of motion. 31 patients were included for analysis totally. Detailed scanning protocols are shown in Figure 1.

For all data acquisition, water suppression was performed using a series of three chemical-shift-selective (CHESS) pulses with predefined flip angles to leave a significant amount of residual water in the spectrum, and high-order shim followed by automatic local shim adjustment was used. Linewidths (Full-Width Half-Maximum (FWHM)) and water suppression were obtained.

### 2.3. Statistical Analysis

The paired *t*-test was used for Comparison (1): comparison of the shimming and water suppression with and without outer volume saturation bands before administration of Gd-DTPA; Comparison (2): comparison of the shimming and water suppression with and without outer volume saturation bands after administration of Gd-DTPA; Comparison (3): comparison of the shimming and water suppression between unenhanced and enhanced. 

Using Pearson correlation, we determined relationship between FWHM and water suppression of all acquired MRS prescan data. For all tests, a *P* value less than 0.05 was considered to indicate a statistically significant difference. Statistical analyses were performed with SPSS software (version 10.0.1; SPSS, Chicago, IL).

## 3. Results

**Figure 2:**
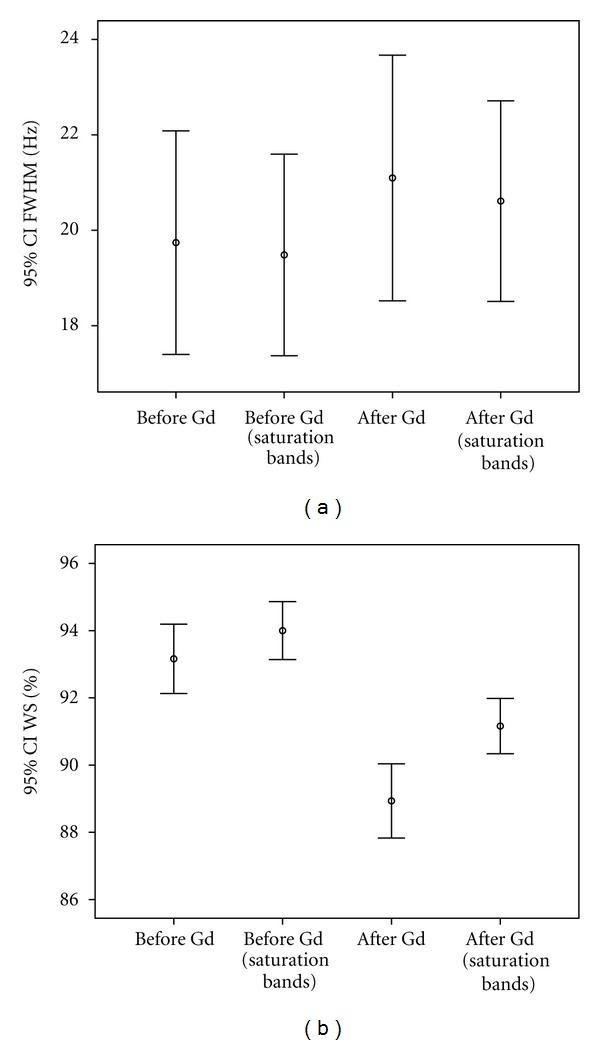
^1^H MRS prescan of shimming (a) and water suppression (b) acquired before and after intravenous administration of Gd-DTPA with and without outer volume saturation bands are shown. After administration of contrast agent, an important point derived from our data is the statistically significant decrease in water suppression and shimming effects. Placement of the outer volume saturation bands is helpful to improve water suppression both before and after contrast agent administration.

The group with the outer volume saturation bands demonstrated slightly better water suppression effect than the group without outer volume saturation bands before administration (94.0 ± 2.4%, 93.2 ± 2.8%, *t* = 3.763, *P* = 0.001). The group with the outer volume saturation bands demonstrated slightly better water suppression effect than the group without outer volume saturation bands after administration (91.2 ± 2.2%, 88.9 ± 3.0%, *t* = 10.811,  *P* < 0.000). Both shimming (20.6 ± 5.7 Hz, 19.5 ± 5.8 Hz, *t* = −2.137, *P* = 0.041) and water suppression effects (91.2 ± 2.2%, 94.0 ± 2.4%, *t* = 8.649, *P* < 0.000) were decreased on enhanced MRS prescan adjustments (Figure 2).

**Figure 3:**
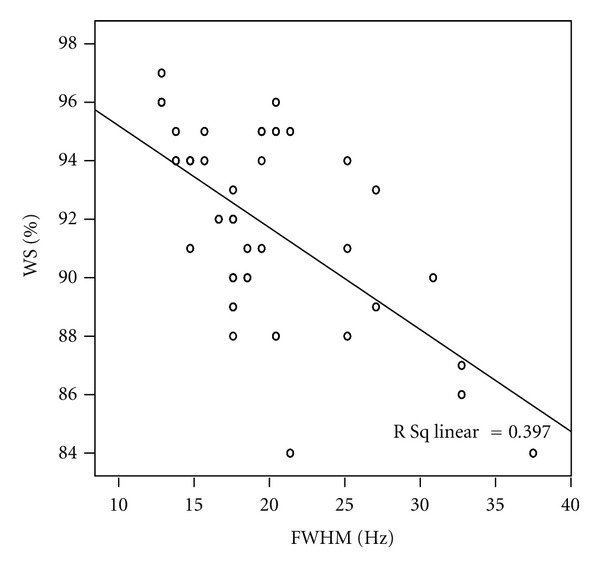
The scatter plots reveal relationships between FWHM and water suppression of all acquired MRS prescan data. A good inverse correlation was observed (*r* = −0.630, *P* = 0.006).

The scatter plots of all acquired data reveal relationship between FWHM and water suppression. We found the line goes from a high-value on the *y*-axis down to a high-value on the *x*-axis suggesting that the variables have anegative correlation (*r* = −0.630, *P* = 0.006) (Figure 3).

## 4. Discussion

Since spectroscopy at 3.0T provides improved SNR and spectral resolution compared with 1.5T MRI scanners, it is expected to yield more reliable measurements of metabolite concentrations [6, 8, 13]. Changes in metabolite signal related to gadolinium contrast administration have been previously reported in many organ systems [11, 12, 14, 15]. However, there were no previous comprehensive reports on hepatic magnetic resonance spectroscopic prescanningon a 3.0-T system. After administration of Gd-DTPA, according to the data from our study, statistically significant decrease in water suppression and shimming effects were noted.

It is well known that MRS is more sensitive than MRI to nonuniformities in the magnetic field. Shimming is important for all MR applications, but critically important for MRS and T2*-based imaging. In MRS, the line width of a peak is dependent both on the intrinsic T2 of that metabolite and the homogeneity of the magnetic field in the region. The line width of a peak because of its intrinsic T2 is typically less than 1 Hz, whereas the line width from field inhomogeneity may be much larger [16, 17]. In a previous study, two spectra were obtained subsequently from the same ROI in the same subject with intentionally decreased shim quality, and Kreis [18] found that all distinct features of the Glu/Gln region are lost with bad linewidth with larger FWHM. 

What are the potential causes for the different water suppression and shimming after contrast media injection? Theoretical and experimental evidence suggests that spectroscopic data, or metabolic measurements, may be affected by Gd-DTPA [11]. Contrast agents such as Gd-DTPA cause T1 shortening once they leak into the interstitial space. On the other hand, contrast agents that remain in the vascular space cause a signal intensity decrease as a result of the increased inhomogeneity of local magnetic fields (T2* shortening effect). Water suppression techniques for *in vivo* proton MRS are based on exploiting a suitable physical parameter distinguishing the protons of water from those of the observed metabolites. Water suppression efficiency achieved with the CHESS techniques is always affected by inhomogeneities of the exciting B1 field [9, 19]. Owing to T2* shortening effect, the homogeneous magnetic environment becomes hard to achieve. And small errors in the pulse amplitudes can result in poor water suppression [9, 19].

In the study of the nervous system, some authors view these effects of Gd-DTPA as clinically unimportant [11, 14]. Joe and coworkers [12] reported all shim current values were the same for pre- and postcontrast measurements on a total of 25 measurements of breast cancer except for 2. These spectra in nervous system and breast were obtained between 10 and 20 minutes after contrast administration. Our study showed significant shimming and water suppression effects of Gd-DTPA on the spectra of liver. This result is not surprising. The spectra in our patients were obtained between 120 and 160 seconds after contrast administration, when the magnetic susceptibility effects would probably be significantly larger.

What are the potential causes for the different water suppression with and without outer volume saturation bands? Spatial suppression of peripheral regions (outer volume suppression) is used in MR spectroscopic imaging to reduce contamination from strong lipid and water signals [20]. The effect of outer volume saturation bands on signal homogeneity in MR 2D chemical shift imaging has been assessed and Wu and coworkers [21] found that outer volume saturation bands improve spatial signal homogeneity. We also noted an approximately 0.2 Hz precontrast and an approximately 0.5 Hz postcontrast decrease of FWHM with outer volume saturation bands (although no significant changes were found due to small sample size). These results are consistent with the theoretical model. When there is worse shimming, then determined reference frequency is less correct. And small errors in the pulse amplitudes can result in uneffective water suppression. The correlation analyses of all acquired data of FWHM and water suppression also confirm a significant negative correlation.

This technique has its limitations in methodology. *In vivo* hepatic MRS studies focus on the status of the liver as a whole or on the characterization of focal lesions. In the latter category of MRS studies one obviously wants to be able to measure MRS voxels smaller. High-field MRI equipment and/or advanced techniques, such as nuclear Overhauser effect enhancement and proton decoupling, may demonstrate improved signal-to-noise ratios and spectral resolution between MRS peaks. The application of those new techniques may be necessary to answer this question. 

In conclusion, placement of the outer volume saturation bands is helpful to improve water suppression both before and after contrast agent administration. Gd-DTPA exerts a slightly adverse effect (a statistically significant butclinically unimportant) in MRS prescan adjustments at 3T. It means that contrast material may be administered before clinical hepatic MR spectroscopy. Knowledge of these findings is helpful to clinician.

This paper will establish a solidfoundation to investigations on further research on the difference of spectral pattern of the liver. Further study should be continued to obtain the detail data by increasing patients.
